# Wechselwirkungen in der dermatologischen Systemtherapie

**DOI:** 10.1007/s00105-020-04726-9

**Published:** 2020-11-26

**Authors:** Kristina Krause, Katharina Jahn, Bernhard Homey

**Affiliations:** Klinik für Dermatologie, Universitätsklinikum Düsseldorf, Moorenstr. 5, 40225 Düsseldorf, Deutschland

**Keywords:** Arzneimittelnebenwirkungen, Krankenhauseinweisung, Unerwünschte Arzneimittelwirkungen, Medikamentenwechselwirkungen, Polypharmazie, Drug side effects, Hospitalization, Adverse drug reactions, Drug interactions, Polypharmacy

## Abstract

Schwere Arzneimittelnebenwirkungen stellen mit 5–7 % einen häufigen Grund für eine Krankenhauseinweisung dar. Die Prävalenz von unerwünschten Arzneimittelwirkungen (UAW) während eines stationären Aufenthaltes liegt sogar bei etwa 11,5 %. Die Ursache sind oftmals Medikamentenwechselwirkungen, bedingt durch die Polypharmazie der multimorbiden älteren Patienten. So nimmt ein 65-jähriger Patient im Durchschnitt 5 Medikamente gleichzeitig ein. Aufgrund des zunehmenden Einsatzes von Systemtherapeutika in der Dermatologie und der gleichzeitig zunehmenden Polypharmazie ist das Wissen um Medikamenteninteraktionen für den Dermatologen zur Vermeidung schwerer Arzneimittelnebenwirkungen wesentlich. Dieser Beitrag soll eine Hilfestellung bieten, Patienten und Medikamente mit einem hohen Risiko für schwerwiegende Interaktionen zu identifizieren und dadurch das Auftreten unerwünschter Wirkungen oder auch die Verminderung des therapeutischen Effektes von Wirkstoffen zu vermeiden. Wir möchten darauf hinweisen, dass die folgende Arbeit einzelne Aspekte behandelt und nicht von der Überprüfung einzelner Arzneimittelinteraktionen mittels Interaktionsprogrammen entbindet. Nicht außer Acht gelassen werden sollte, dass neben verschreibungspflichtigen Medikamenten auch Nahrungsmittel, Nahrungsergänzungsmittel und Kräuter zu Interaktionen mit Medikamenten führen können.

**Zusatzmaterial online:**

Die Online-Version dieses Beitrags (10.1007/s00105-020-04726-9) enthält zusätzlich 12 Abbildungen. Beitrag und Zusatzmaterial stehen Ihnen im elektronischen Volltextarchiv auf https://www.springermedizin.de/der-hautarzt zur Verfügung. Sie finden das Zusatzmaterial am Beitragsende unter „Supplementary Material“.

## Abstract (en)

Severe pharmacological side effects have an occurrence of 5–7% and represent a frequent reason for hospital admission. The prevalence of undesired pharmacological side effects during hospitalization is even higher with approximately 11.5%. The causes are often interactions between drugs due to the polypharmacy of multimorbid older patients. On average, a 65-year-old male patient will simultaneously be taking 5 medications. Due to the increasing use of systemic drugs in dermatology and the simultaneously increasing polypharmacy, knowledge of interactions between medications is essential for dermatologists in order to avoid severe side effects of drugs. This article provides assistance in order to identify patients and medications with a high risk for severe interactions and, therefore, to avoid the occurrence of undesired effects or the reduction of the therapeutic effects of active substances. We would like to point out that this article deals with individual aspects and does not mean that the testing of individual drug interactions with interaction programs can be omitted. It should also not be neglected that in addition to prescription-only drugs, foodstuffs, dietary supplements and herbs can also lead to interactions with medications.

Grundsätzlich werden in der Pharmakologie pharmakodynamische und -kinetische Wechselwirkungen unterschieden. Dabei stellt die Pharmakodynamik die Analyse der biologischen Wirkung eines Pharmakons dar und beschreibt den Einfluss von Arzneimitteln auf den Organismus.

Pharmakokinetische Interaktionen beziehen sich auf Prozesse, die ein Arzneimittel im Körper durchläuft (Online-Abb. 1).

Das Risiko von Medikamenteninteraktionen steigt nicht nur durch die Polypharmazie, sondern auch durch Funktionsstörungen wichtiger Organe wie Leber, Nieren und Herzfunktion sowie durch eingeschränkte Stoffwechselleistungen bei Adipositas, Hypothyreose und Hypoproteinämie [1–4]. Genetische Faktoren können ebenfalls die Metabolisierung von Medikamenten beeinflussen (Online-Abb. 2). So kommt es beispielsweise durch einen angeborenen Mangel des Enzyms Thiopurine-Methyltransferase (TPMT) zur Akkumulation von Abbauprodukten von Azathioprin und damit zu vermehrten Nebenwirkungen. Durch den genetischen Polymorphismus am NAT2-Lokus kommt es zur Ausprägung eines langsamen und eines schnellen Acetylator-Phänotyps der Arylamin-N-Acetyltransferase 2, die unter anderem Medikamente wie Isoniazid, Hydralazin und Dapson verstoffwechselt. So geht eine reduzierte Metabolisierung von Isoniazid mit deutlich mehr hepatotoxischen Nebenwirkungen einher. Der genetische Polymorphismus des Enzyms CYP2D6, das am Stoffwechsel etwa jedes vierten Arzneimittels beteiligt ist, führt bei einer genetisch bedingten Defizienz zu einer deutlich verlangsamten Elimination des Arzneimittels aus dem Körper, was eine relative Überdosierung mit entsprechend verstärkten Nebenwirkungen bedingt [5]. Eine Bestimmung der TPMT-Aktivität ist im Rahmen einer Therapie mit Azathioprin unerlässlich.

## Expertentipp.

Um eine Verzögerung des Therapiebeginns mit Azathioprin bis zum Eingang der TPMT-Aktivität zu umgehen, empfehlen wir am Tag der Abnahme eine dosisreduzierte Einleitung von Azathioprin und eine ergebnisabhängige Anpassung im Verlauf.

Eine weitere genetische Prädisposition mit hoher Relevanz für den klinischen Alltag stellt der Glucose-6-Phosphat-Dehydrogenase-Mangel dar. Dabei handelt es sich um eine Mutation im x‑chromosomalen *G6PD*-Gen, wodurch es zu einer erhöhten Anfälligkeit der Erythrozytenmembran für freie Sauerstoffradikale kommt. Bei erhöhtem oxidativem Stress, z. B. medikamentös bedingt durch eine Therapie mit Dapson, kann es bei Vorliegen eines Glucose-6-Phosphat-Dehydrogenase-Mangels zu einer Zerstörung der Erythrozyten bis hin zur hämolytischen Krise kommen [16].

## Pharmakokinetische Interaktionen

Die Pharmakokinetik beschreibt den Einfluss des Organismus auf die Arzneistoffe. Im Wesentlichen geht es um diejenigen Vorgänge, die nach der Gabe eines Medikamentes in den Verteilungsräumen des Körpers ablaufen. Hierzu zählen Resorption, Distribution, biochemische Um- und Abbauprozesse (Metabolisierung) sowie Elimination.

Bei der gleichzeitigen Verabreichung mehrerer Arzneimittel kann es zu einer gegenseitigen Beeinflussung der pharmakokinetischen Prozesse und einer Änderung konzentrationsabhängiger Wirkungen kommen.

### Lebermetabolismus

**Figure 1:**
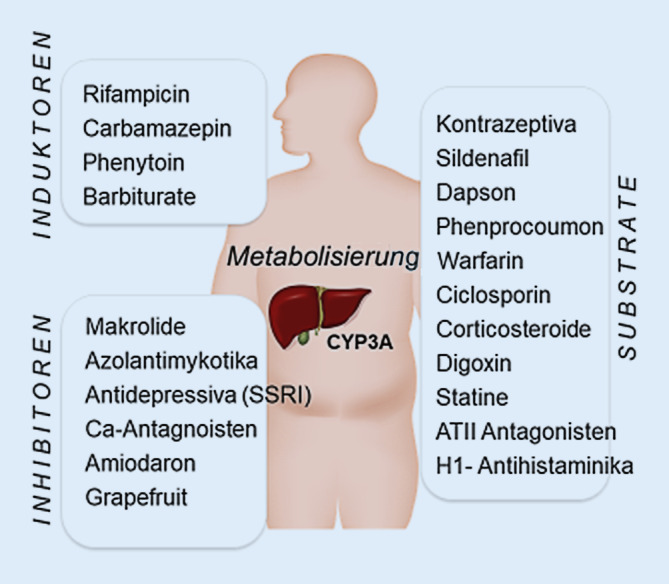

Die meisten pharmakokinetischen Interaktionen beruhen auf einer veränderten Metabolisierung in der Leber durch Induktion oder Inhibition der Cytochrom-P450(CYP)-Isoenzyme. Mehr als die Hälfte dieser Reaktionen finden über das Isoenzym CYP 3A4 statt. Die häufigsten Induktoren, Inhibitoren und Substrate sind in Abb. 1 zusammengefasst, wobei sowohl Inhibitoren als auch Induktoren ebenfalls als Substrate der CYP-Enzyme fungieren können. Hierbei ist insbesondere auf Medikamente mit einer geringen therapeutischen Breite zu achten, wie beispielsweise Ciclosporin, Digoxin, Phenytoin, Dapson und Warfarin, deren verringerte oder vermehrte Metabolisierung zu lebensbedrohlichen Komplikationen führen kann. Zu den CYP-3A4-Induktoren zählt unter anderen auch Johanniskraut, das von Patienten oft nicht als Medikament verstanden und entsprechend oft in der Arzneimittelanamnese nicht erfasst wird.

Die in der Dermatologie häufig eingesetzten Azol-Antimykotika (Itraconazol, Fluconazol und Ketoconazol) gehören zu den stärksten Inhibitoren des Isoenzyms CYP 3A4 und führen somit zu einer verminderten Metabolisierung und einem erhöhten Plasmaspiegel anderer CYP-Substrate. So muss der Wirkspiegel von Substraten wie Ciclosporin, Warfarin, Cyclophosphamid und Digoxin strengstens kontrolliert werden, um lebensbedrohliche Komplikationen wie Blutungen unter erhöhten Warfarin-Plasmaspiegeln zu vermeiden. Des Weiteren verursacht beispielsweise das Antimykotikum Terbinafin durch seine Inhibition des Isoenzyms CYP 2D6 eine reduzierte Metabolisierung von insbesondere β‑Blockern, deren erhöhte Plasmaspiegel bedrohliche Bradykardien hervorrufen können (Online-Abb. 3).

Dosisabhängige unerwünschte bis lebensbedrohliche Wirkungen einer immunsuppressiven Therapie mit Azathioprin, das unter anderem zur Behandlung des Lupus erythematodes und autoimmuner blasenbildender Dermatosen zugelassen ist, sind durch eine Suppression des Knochenmarks bedingte Leukopenie, Anämie oder Thrombozytopenie sowie in seltenen Fällen eine Agranulozytose und Panzytopenie. Im menschlichen Körper wird Azathioprin durch das Enzym Glutathion-S-Transferase zu dem aktiven Metaboliten 6‑Mercaptopurin metabolisiert. Dieser interferiert als atypisches Nukleosid mit der DNA(Desoxyribonukleinsäure)‑/RNA(Ribonukleinsäure)-Synthese und wird durch das Enzym Thiopurinmethyltransferase und andere Enzyme wie die Xanthinoxidase weiter verstoffwechselt. Die gleichzeitige Gabe des Urikostatikums Allopurinol verhindert durch die Hemmung der Xanthinoxidase den Abbau des 6‑Mercaptopurin zu inaktiven Metaboliten (6-Thioharnsäure) und führt dadurch zu einem gesteigerten Plasmalevel des aktiven Metaboliten 6‑Mercaptopurin und damit verbundener gesteigerter Toxizität (Online-Abb. 4). Falls die gleichzeitige Anwendung beider Medikamente nicht vermieden werden kann, sollte daher eine Dosisreduktion von Azathioprin auf etwa 25 % erfolgen.

Auch Nahrungsmittel können zu pharmakokinetischen Interaktionen führen. Die in Zitrusfrüchten (besonders in Grapefruit) enthaltenen Furanocoumarine Bergamottin und 6′,7′-Dihydroxybergamottin sind ebenfalls Hemmstoffe von CYP 3A4 und können die Bioverfügbarkeit einer Vielzahl anderer Arzneistoffe erhöhen [6].

### Absorption

Antibiotika wie beispielsweise Makrolide führen nicht nur über eine Inhibition der CYP-Metabolisierung zu einem erhöhten Plasmawirkspiegel der Vitamin-K-Antagonisten (Phenprocoumon), sondern steigern deren Wirksamkeit und das damit verbundene Blutungsrisiko auch durch eine Reduktion Vitamin-K-produzierender Darmbakterien und einer folglich verminderten Vitamin-K-Resorption (Online-Abb. 5).

### Renale Elimination

Während bei den meisten Interaktionen eine genauere Quantifizierung des Ausmaßes der Interaktion nicht vorhersagbar ist, kann für renal eliminierte Pharmaka in Abhängigkeit von der glomerulären Filtrationsrate (GFR) eine Dosisadaptation berechnet werden. Dies muss insbesondere bei der Gabe von Aciclovir im Rahmen einer Zoster-Erkrankung bei älteren Patienten oder bei vorbekannter Niereninsuffizienz beachtet werden. Dabei sollte eine gleichzeitige Verabreichung nephrotoxischer Medikamente (Ciclosporin, Aminoglykoside, nichtsteroidale Antirheumatika, Penicilline und Cephalosporine) vermieden werden (Online-Abb. 6). Eine Alternative zur Behandlung des Zosters stellt das antivirale Arzneimittel Brivudin dar. Während Brivudin auch bei niereninsuffizienten Patienten angewendet werden kann, besteht für die Behandlung von immunsupprimierten Patienten aufgrund der unzureichenden Studienlage keine Zulassung. Kontraindiziert ist eine Therapie mit Brivudin bei gleichzeitiger Gabe von 5‑Fluoruracil aufgrund einer gesteigerten Toxizität.

Auch bei der Verabreichung von Methotrexat muss sowohl vor als auch während der Therapie auf eine ausreichende Nierenfunktion geachtet werden. Bei bereits eingeschränkter Nierenfunktion sollte Methotrexat mit Vorsicht angewandt und ab einer GFR von <60 ml/min auf die Hälfte der Dosis reduziert werden. Liegt eine stark reduzierte Nierenfunktion (GFR <30 ml/min) vor, darf Methotrexat nicht verabreicht werden [7].

Die Eliminierung von Methotrexat erfolgt renal über glomeruläre Filtration sowie aktive tubuläre Sekretion. Bei eingeschränkter Nierenfunktion und verminderter renaler Elimination kommt es zu einer verlängerten Plasmahalbwertszeit und einer gesteigerten intrazellulären Aufnahme und Akkumulation von Methotrexat [15]. Wird die intrazelluläre Aufnahmekapazität überschritten, kommt es zur Suppression der Replikation von schnell proliferierendem Gewebe. Myelodepression bis hin zu Agranulozytose sind mögliche und schwerwiegende Folgen.

Die Evaluation der GFR erfolgt im klinischen Routinealltag primär über den Kreatininwert im Serum. Dieser ist alters- und geschlechtsabhängig und steigt im Serum erst bei einer Reduktion der GFR auf etwa 50 ml/min (Normwert 130 ml/min). Daraus ergibt sich, dass eine Einschränkung der GFR bis zu diesem Punkt nicht erkannt wird. Cystatin C hingegen wird frei glomerulär filtriert, im proximalen Tubulus komplett reabsorbiert und katabolisiert. Die Konzentration von Cystatin C im Serum wird somit weitestgehend durch die glomeruläre Filtrationsrate (GFR) bedingt und ermöglicht eine höhere diagnostische Genauigkeit.

Gerade bei der Verabreichung mehrerer Medikamente, die allein oder in Kombination Einfluss auf die renale Clearance nehmen können [8], sollte eine zusätzliche Bestimmung der GFR über die Cystatin-C-Serumkonzentration erfolgen. Gegebenenfalls ist eine Umstellung der Medikation von nierenfunktionsabhängigen auf nierenfunktionsunabhängige Medikamente zu empfehlen (Tab. 1).

**Table 1:** 

Gruppe	Nierenfunktionsabhängig	Nierenfunktionsunabhängig
Analgetika	Morphin (M6-Glucuronid), Pethidin (Norpethidin)	Fentanyl, Levomethadon
Antibiotika	Ciprofloxacin, Levofloxacin	Moxifloxacin
Antiarrhythmika	Sotalol	Amiodaron
Antidiabetika	Glibenclamid, Glimepirid (Hydroxymetabolit)	Moxifloxacin
–	Nateglinid	Pioglitazon
Antihypertensiva	Atenolol	–
Antikonvulsiva	Gabapentin, Pregabalin, Lamotrigin, Levetiracetam	Carbamazepin, Valproat
Cholesterinsenker	Bezafibrat, Fenofibrat	Simvastatin, Niazin
Gicht- und Rheumamittel	Methotrexat	Hydroxychloroquin, Leflunomid
Herz-Kreislauf-Mittel	Digoxin	Digitoxin
Psychopharmaka	Lithium, Mirtazapin	Amitriptylin, Citalopram, Haloperidol, Risperidon
Virostatika	Aciclovir	Brivudin

**Table 2:** 

Gruppe	Substanzen	Interaktionen	Komplikationen
Antimykotika	Itraconazol	Phenprocoumon	Erhöhtes Blutungsrisiko
	Ketoconazol		
Antibiotika	Ciprofloxacin	Phenprocoumon	Erhöhtes Blutungsrisiko
	Clindamycin		
	Tetrazyklin		
Antikonvulsiva	Gabapentin	Morphin	Somnolenz
	Pregabalin		
Gicht- und Rheumamittel	Methotrexat	Ibuprofen	Thrombembolische Komplikationen
Glukokortikoide	Prednisolon	NSAR	Gastrointestinale Blutung, Magenulzera
Immunsuppressiva	Azathioprin	Allopurinol	Anämie, Leukopenie, Leberwerterhöhung
	Ciclosporin	Statine	Rhabdomyolyse
Malariamittel	Hydroxychloroquin	Amiodaron	QT-Intervall-Verlängerung
		Sotalol	
		Fluorchinolone	
		Makrolide	
		Dabrafenib	
		Vemurafenib	
Retinoide	Isotretinoin	Statine	Rhabdomyolyse
	Acitretin	Fibrate	
	Alitretinoin		
	Bexaroten	Tetrazyklin	Pseudotumor cerebri
Virostatika	Aciclovir	Ciclosporin	Niereninsuffizienz
		Aminoglykoside	
		NSAR	
		Penicilline	
		Cephalosporine	

*NSAR* nichtsteroidales Antirheumatikum

## Pharmakodynamische Interaktionen

In der Pharmakodynamik, die das Wirkprofil eines Medikaments am Zielort beschreibt, können additive bzw. synergistische und antagonistische Wechselwirkungen von Medikamenten unterschieden werden. Ein synergistischer Effekt ist oftmals erwünscht und findet in Form von Kombinationstherapien unter anderem in der Schmerztherapie nach WHO(World Health Organization)-Stufenschema, bei der Behandlung mit Antiinfektiva, in der Tumortherapie oder bei der Behandlung der Hypertonie Anwendung. Eine synergistische Wirkung kann jedoch auch unerwünschte Komplikationen hervorrufen. So besteht beispielsweise durch die gleichzeitige Gabe von Methotrexat und Sulfonamiden das erhöhte Risiko des Auftretens einer Panzytopenie infolge einer Knochenmarksuppression bedingt durch einen additiven Folsäureantagonismus [9].

## QT-Zeit-Verlängerung

Sowohl pharmakodynamische als auch pharmakokinetische Arzneimittelinteraktionen können eine QT-Zeit-Verlängerung bedingen. Zudem gibt es viele Faktoren, die für eine Verlängerung der QT-Zeit prädisponieren wie beispielsweise fortgeschrittenes Alter, weibliches Geschlecht, linksventrikuläre Herzinsuffizienz, Diabetes mellitus, Bluthochdruck, Hyperthyreose und Elektrolytstörungen (Hypokaliämie und Hypomagnesiämie) [10]. Allerdings ist eine der häufigsten Ursachen für eine erworbene QT-Zeit-Verlängerung die Einnahme von Medikamenten, die eine Blockierung der schnellen Komponente des verzögerten Gleichrichter-Kalium-Kanals bewirken und somit zu malignen Herzrhythmusstörungen wie Torsade de pointes führen können [11].

Die pharmakodynamische Wechselwirkung beruht dabei unter anderem auf einem synergistischen Effekt von Medikamenten wie Chloroquin und Hydroxychloroquin und ohnehin QT-Zeit-verlängernden Medikamenten wie den Makrolidantibiotika (Clarithromycin und Erythromycin), Fluorchinolonen, Cotrimoxazol, Azolantimykotika, Amiodaron, BRAF-Inhibitoren (Dabrafenib und Vemurafenib), Serotonin-Reuptake-Inhibitoren und trizyklischen Antidepressiva (Online-Abb. 7).

Ein pharmakokinetischer Effekt kann auftreten, wenn die Plasma- und Gewebekonzentrationen eines QT-Zeit-verlängernden Medikaments erhöht sind, beispielswiese durch eine reduzierte Metabolisierung der Medikamente (s. CYP-450-Inhibitoren, Abb. 1).

## Risikoreiche Medikamentengruppen

Einige Medikamentengruppen gelten als besonders risikoreich. Neben der Gefahr von zum Teil schwerwiegenden Nebenwirkungen kann es zu zahlreichen pharmakodynamischen wie auch -kinetischen Medikamenteninteraktionen kommen.

### Orale Retinoide

Bei der Therapie mit oralen Retinoiden müssen mehrere unerwünschte Interaktionen bedacht werden. Die gleichzeitige Gabe von häufig in der dermatologischen Praxis insbesondere zur Therapie der mittelschweren bis schweren Acne vulgaris verordneten oralen Retinoiden mit Tetrazyklinen erhöht das Risiko einer intrakraniellen Druckerhöhung im Sinne eines Pseudotumors cerebri und stellt somit eine absolute Kontraindikation dar.

Des Weiteren muss unter der Therapie mit systemischen Retinoiden wie Isotretinoin, Alitretinoin, Acitretin oder Bexaroten auf das seltene, aber potenzielle Risiko einer lebensbedrohlichen Rhabdomyolyse geachtet werden [12]. Dieses Risiko ist insbesondere durch gleichzeitige Gabe lipidsenkender Medikamente wie Statine und Fibrate erhöht, die oftmals aufgrund des Anstiegs der Lipide, insbesondere der Triglyzeride, unter einer Retinoidtherapie erfolgt (Online-Abb. 8). Aufgrund dessen müssen bei einer Therapie mit Retinoiden neben Leber- und Lipidwerten auch die Muskelenzyme (CK) regelmäßig laborchemisch kontrolliert werden. Die Patienten sollten darauf hingewiesen werden, starke körperliche Belastungen (beispielsweise Leistungssport) unter der Therapie zu vermeiden und sich bei Auftreten von Muskelschmerzen umgehend bei ihrem behandelnden Arzt vorzustellen (Online-Abb. 9).

### Azole

Das hohe pharmakokinetische Interaktionspotenzial der Azolantimykotika (Fluconazol, Isavuconazol, Itraconazol, Posaconazol und Voriconazol) ist v. a. durch die Verstoffwechselung über das Isoenzym Cytochrom P450 bedingt [14]. Kommen Azole in einer Kombinationstherapie zur Anwendung, ist die Verwendung von Interaktionsprogrammen unerlässlich.

### Immunsuppressiva

Besondere Achtsamkeit ist bei der Anwendung systemischer Immunsuppressiva angebracht, da durch Medikamenteninteraktionen verursachte erhöhte Plasmaspiegel zu einer gesteigerten Toxizität und einer Suppression des Knochenmarks führen können mit daraus resultierenden lebensbedrohlichen Komplikationen, bedingt durch eine Panzytopenie und potenzielle infektiöse Komplikationen. Unter einer Therapie mit Methotrexat (MTX) sollte insbesondere auf eine gesteigerte Lebertoxizität und die Suppression des Knochenmarks geachtet werden.

Nichtsteroidale Antirheumatika (NSAR), die für gewöhnlich auch zur Behandlung akuter oder chronischer Schmerzen wie bei der Psoriasisarthritis eingesetzt werden, erhöhen die Serumkonzentration von MTX und damit unter anderem das Risiko für aplastische Anämien, Knochenmarksuppression und gastrointestinale Toxizität. Eine engmaschige Kontrolle der Laborwerte sowie die Sensibilisierung des Patienten für das mögliche Auftreten unerwünschter Ereignisse sollten bei gleichzeitiger Gabe von MTX und NSAR erfolgen (Online-Abb. 10).

Unter einer Ciclosporin-Therapie sind aufgrund der geringen therapeutischen Breite des Medikaments sowie der durch CYP-Induktoren und -Inhibitoren beeinflussten Metabolisierung (s. Abb. 1) regelmäßige Plasmaspiegelbestimmungen zur Verhinderung toxischer Nebenwirkungen sinnvoll. Insbesondere die gleichzeitige Gabe anderer nephrotoxischer, zytotoxischer oder immunsuppressiver Arzneimittel erfordert ein genaues Monitoring und ggf. eine Anpassung der Ciclosporin-Dosis.

Protonenpumpeninhibitoren (PPI), die in großem Umfang und oftmals über einen langen Zeitraum für die Behandlung von Magensäure-bedingten Erkrankungen eingesetzt werden, erhöhen das Risiko für klinisch signifikante Arzneimittelinteraktionen bei Patienten, die gleichzeitig weitere Arzneimittel einnehmen. Die Verabreichung von PPI erhöht den intragastrischen pH-Wert, wodurch die Hydrolyse von Mycophenolat-Mofetil verlangsamt wird, was wiederum zu einer verringerten Resorption und maximalen Verfügbarkeit von Mycophenolsäure führt (Online-Abb. 11).

Zu den primär bei therapierefraktärer Psoriasis angewandten Biologika (TNF[Tumor-Nekrose-Faktor]-α-Inhibitoren und IL[Interleukin]-12/23-Inhibitoren) gibt es bisher wenige Informationen über beschriebene Arzneimittelinteraktionen. Dennoch wird zur Vermeidung des theoretischen Risikos einer additiven Immunsuppression sowie einer infektiösen Komplikation zur Vorsicht bei der Kombination mit anderen immunsuppressiven Medikamenten geraten.

Impfungen mit Lebendimpfstoffen sollten unter immunsuppressiver Therapie zurückgestellt werden und frühestens 3 Monate nach Absetzten des Immunsuppressivums gegeben werden. Auch die Wirksamkeit anderer Impfungen kann unter Umständen unter der immunsuppressiven Therapie verringert sein.

Ein erhöhtes Risiko für die Entstehung von nichtmelanozytärem Hautkrebs (NMSC) zählt zum Nebenwirkungsprofil von zahlreichen Immunsuppressiva wie Ciclosporin und Azathioprin. Pharmakoepidemiologische Studien haben jüngst auch für die Exposition mit steigenden kumulativen Dosen von Hydrochlorothiazid ein erhöhtes Risiko für die Entstehung von nichtmelanozytärem Hautkrebs gezeigt [13]. Bei Patienten unter immunsuppressiver Therapie sollte aus dermatologischer Sicht die Kombination von immunsupprimierenden Medikamenten mit anderen NMSC entstehungsfördernden Medikamenten gemieden werden. Im Hinblick auf eine gleichzeitige antidiuretische Therapie mit Hydrochlorothiazid sollte eine vorsichtige Risiko-Nutzen-Abwägung erfolgen und ggf. auf alternative Medikamente umgestellt werden.

## Fazit für die Praxis

- Arzneimittelkombinationen sind häufig und müssen kritisch wahrgenommen werden (Online-Abb. 12).
- Mit der Anzahl gleichzeitig eingenommener Arzneimittel steigt auch das Potenzial für unerwünschte Wechselwirkungen – insbesondere in höherem Lebensalter und bei chronischen Erkrankungen (Tab. 2).
- Kenntnisse über die wichtigsten Prinzipien pharmakokinetischer und pharmakodynamischer Arzneimittelinteraktionen sind daher von besonderer Bedeutung. In diesem Zusammenhang ist insbesondere die Metabolisierung über das Cytochrom-P450-System wichtig, da zahlreiche Arzneimittel überwiegend über diese Enzyme verstoffwechselt werden.
- Klinische Konsequenzen zur Vermeidung unerwünschter und teils lebensbedrohlicher Wechselwirkungen sind je nach Arzneimittelkombinationen eine entsprechende Dosisanpassung, eine zeitlich versetzte Einnahme der Medikamente oder auch ein Wechsel bzw. Absetzen von Wirkstoffen.
- Programme zur Überprüfung von Medikamenteninteraktionen sind im klinischen Alltag unverzichtbar.

## Caption Electronic Supplementary Material


