# Supplementary material for: Drug interactions in dermatological systemic treatment
Source: Hautarzt. 2020 Nov 26;72(1):44–9. [Article in German] doi: 10.1007/s00105-020-04726-9 (PMC7803714; doi:10.1007/s00105-020-04726-9)
Supplement: Supplementary file 1 [file 105_2020_4726_MOESM1_ESM.pdf]

Online-Abbildungen 1-12: Wechselwirkungen in der dermatologischen Systemtherapie

Online-Abb. 1 Übersicht Medikamenteninteraktionen

Online-Abb. 2 Faktoren, die das Risiko von Medikamenteninteraktionen beeinflussen

Online-Abb. 3 Lebermetabolismus: CYP-induzierte Interaktion von Azol-Antimykotika und Phenprocoumon

Online-Abb. 4 Lebermetabolismus: Interaktion von Azathioprin und Allopurinol

Online-Abb. 5 Absorption: Interaktion von Antibiotika und Phenprocoumon

Online-Abb. 6 Renale Elimination: Interaktion von Aciclovir mit nephrotoxischen Medikamenten

Online-Abb. 7 QT-Zeit-Verlängerung: Synergistischer Effekt von Chloroquin und QT-Zeit verlängernden Medikamenten

Online-Abb. 8 und 9 Orale Retinoide: Interaktion von orale Retinoiden und Lipidsenkern

Online-Abb. 10 Immunsuppressiva: Interaktionn von Methotrexat (MTX) und nicht-steroidalen Antirheumatika (NSAR)

Online-Abb. 11 Immunsuppressiva: Interaktion von Mycophenolatmofetil und Protonenpumpeninhibitoren

Online-Abb. 12 Polypharmazie im Alter

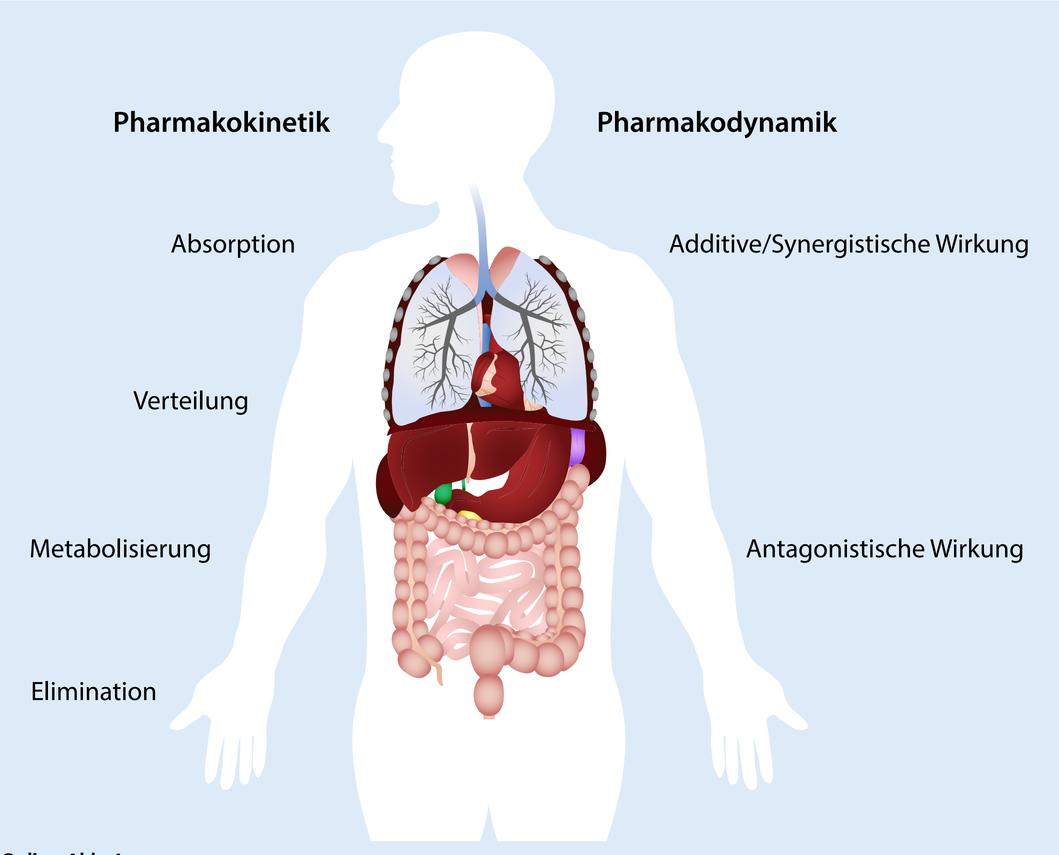
Pharmakokinetik
Pharmakodynamik
Absorption
Additive/Synergistische Wirkung
Verteilung
Metabolisierung
Antagonistische Wirkung
Elimination

Online-Abb. 1

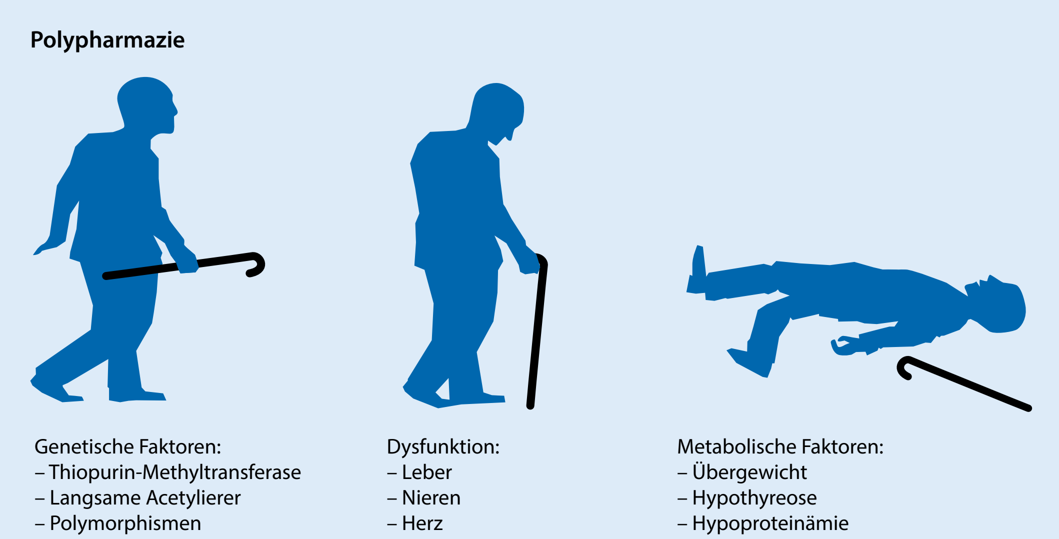
Polypharmazie
Genetische Faktoren:
– Thiopurin-Methyltransferase
– Langsame Acetylierer
– Polymorphismen
Dysfunktion:
– Leber
– Nieren
– Herz
Metabolische Faktoren:
– Übergewicht
– Hypothyreose
– Hypoproteinämie

Online-Abb. 2

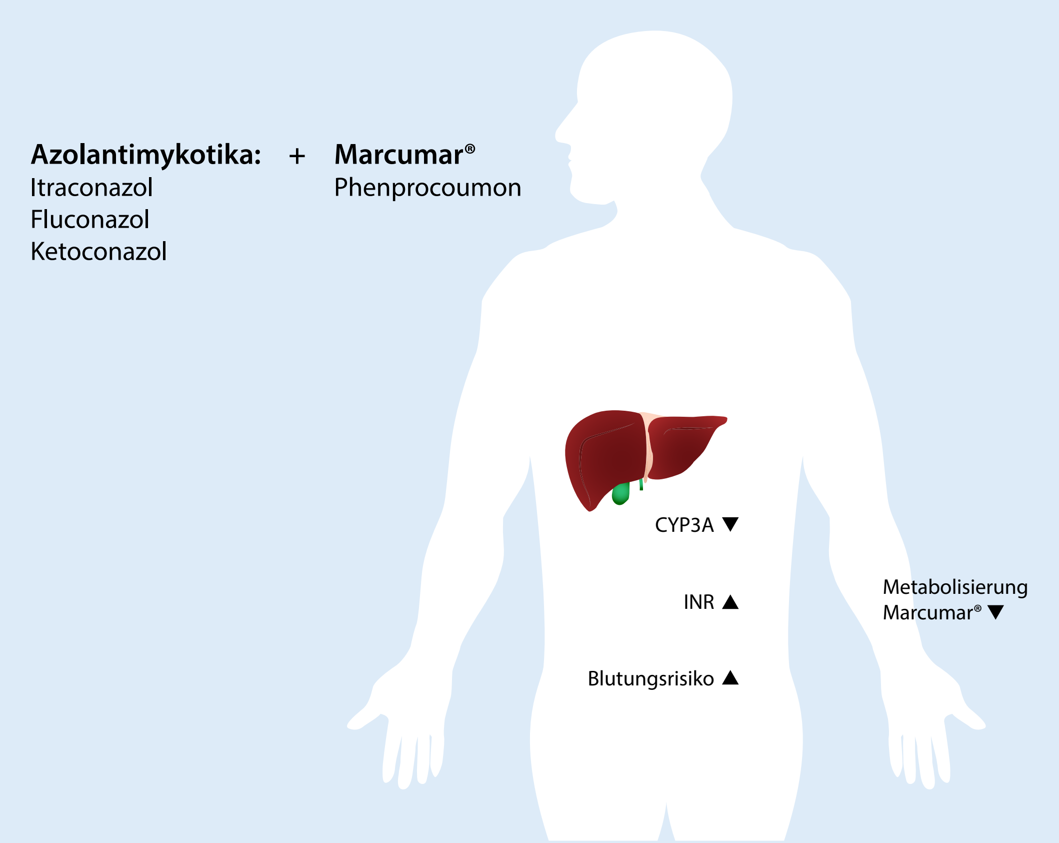
Azolantimykotika:
Itraconazol
Fluconazol
Ketoconazol
+
Marcumar®
Phenprocoumon
CYP3A ▼
INR ▲
Blutungsrisiko ▲
Metabolisierung Marcumar® ▼

Online-Abb. 3

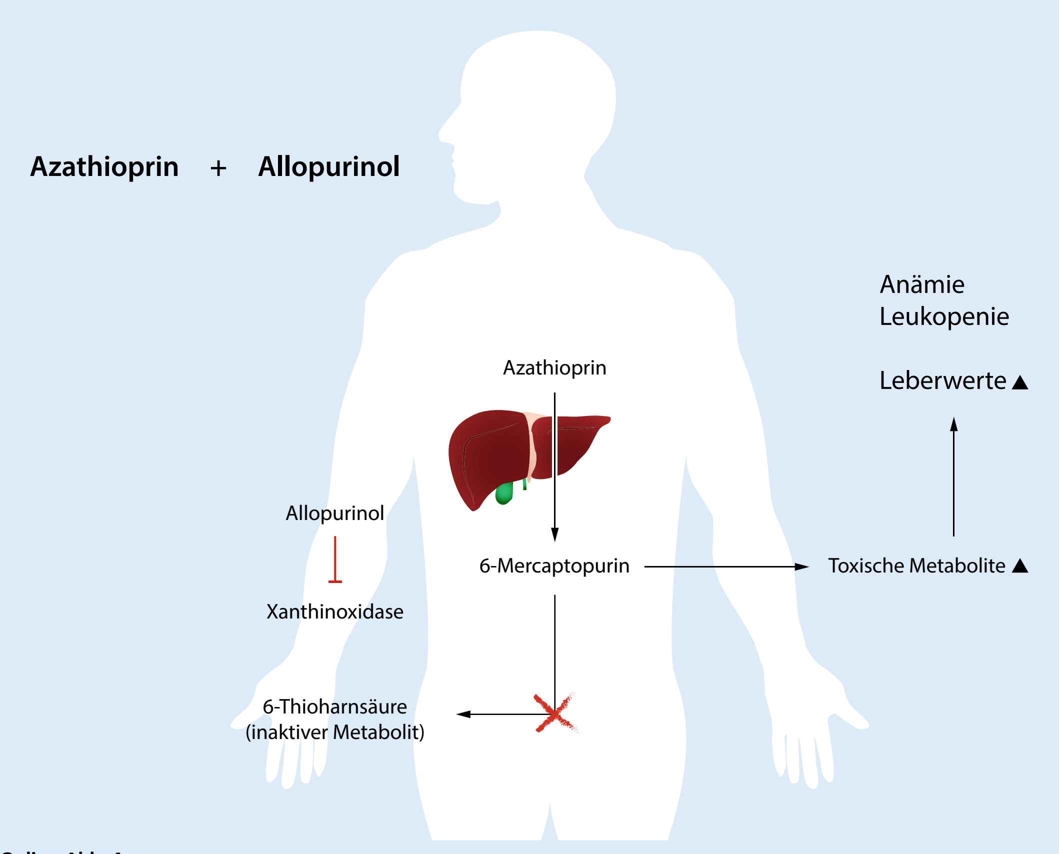
Azathioprin + Allopurinol
Anämie
Leukopenie
Azathioprin
Leberwerte ▲
Allopurinol
6-Mercaptopurin
Toxische Metabolite ▲
Xanthinoxidase
6-Thioharnsäure
(inaktiver Metabolit)

Online-Abb. 4

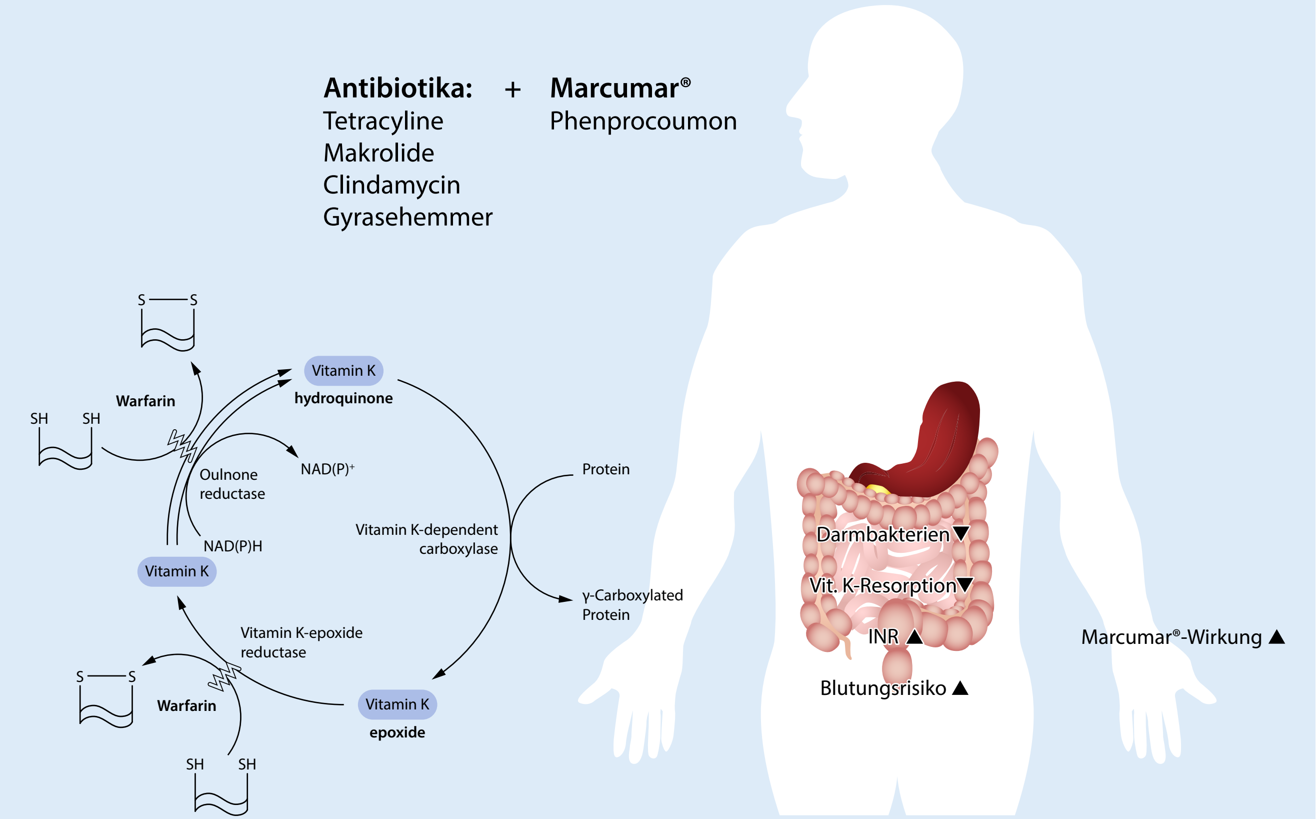
Antibiotika:
Tetracyline
Makrolide
Clindamycin
Gyrasehemmer
+
Marcumar®
Phenprocoumon
S S
SH SH
Warfarin
Vitamin K
hydroquinone
NAD(P)+
Oulnone
reductase
NAD(P)H
Vitamin K
Vitamin K-dependent
carboxylase
Protein
γ-Carboxylated
Protein
Vitamin K-epoxide
reductase
S S
Warfarin
SH SH
Vitamin K
epoxide
Darmbakterien ▼
Vit. K-Resorption ▼
INR ▲
Blutungsrisiko ▲
Marcumar®-Wirkung ▲

Online-Abb. 5

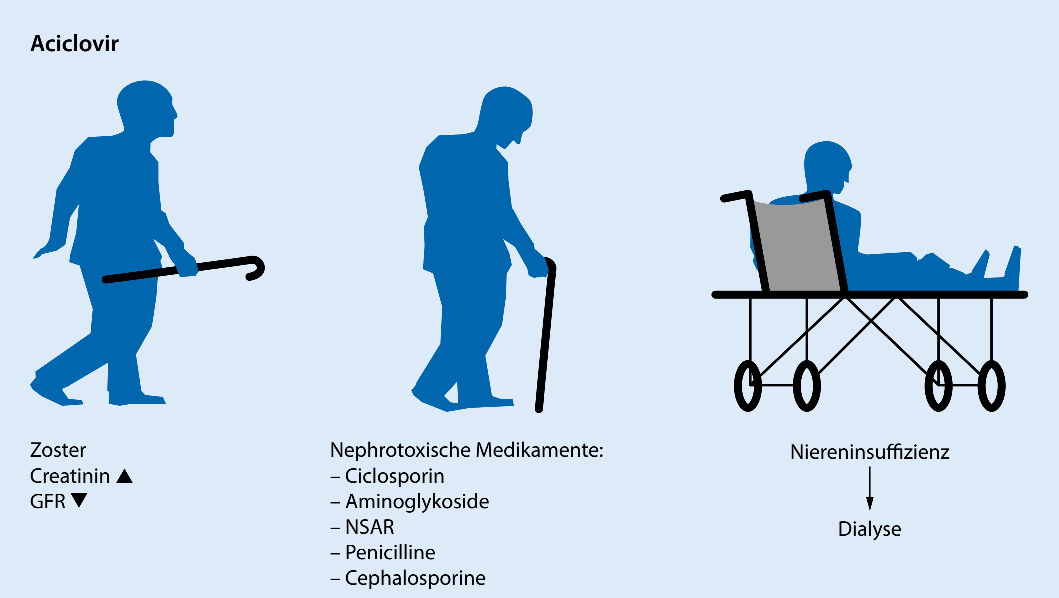
Aciclovir
Zoster
Creatinin ▲
GFR ▼
Nephrotoxische Medikamente:
– Ciclosporin
– Aminoglykoside
– NSAR
– Penicilline
– Cephalosporine
Niereninsuffizienz
Dialyse

Online-Abb. 6

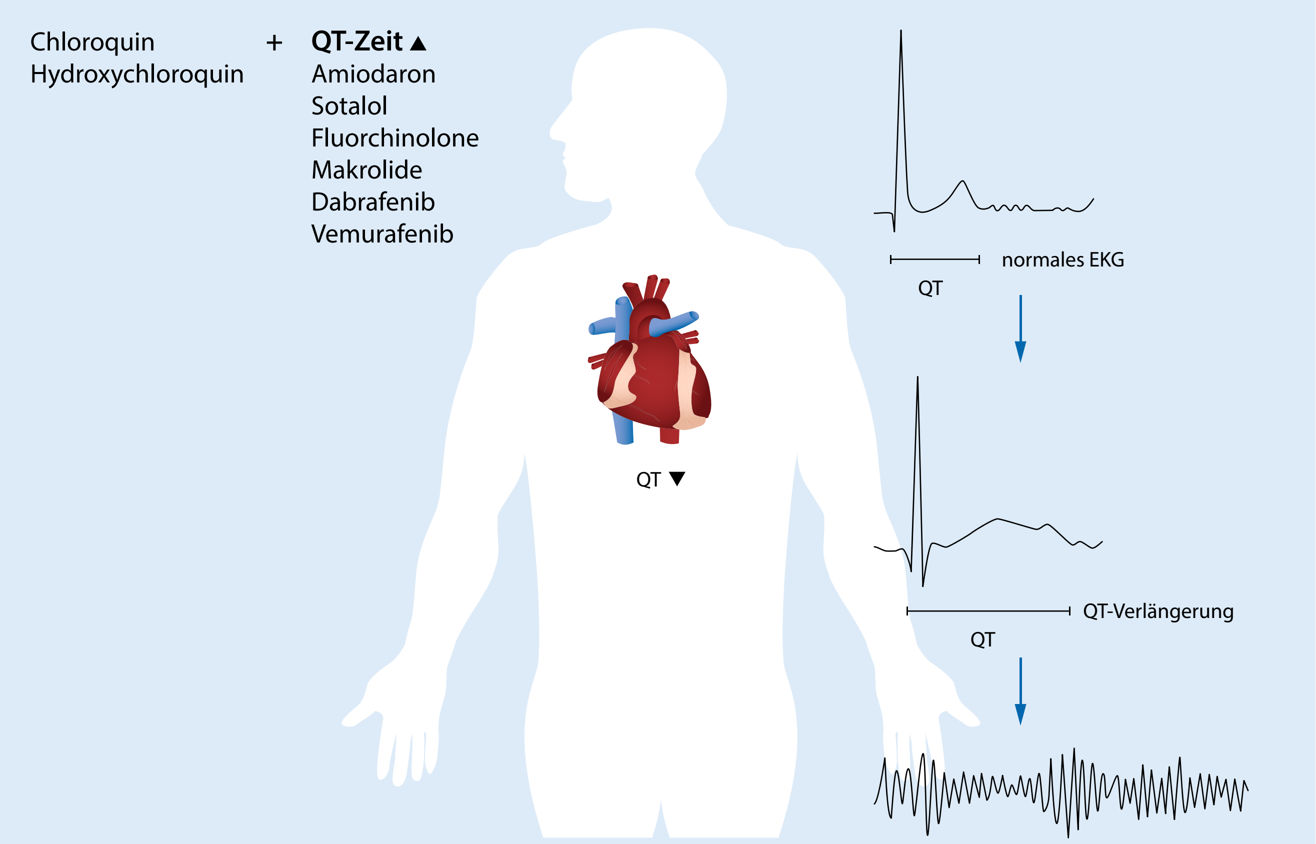
Chloroquin
Hydroxychloroquin
+
QT-Zeit ▲
Amiodaron
Sotalol
Fluorchinolone
Makrolide
Dabrafenib
Vemurafenib
QT ▼
normales EKG
QT
QT-Verlängerung
QT

Online-Abb. 7

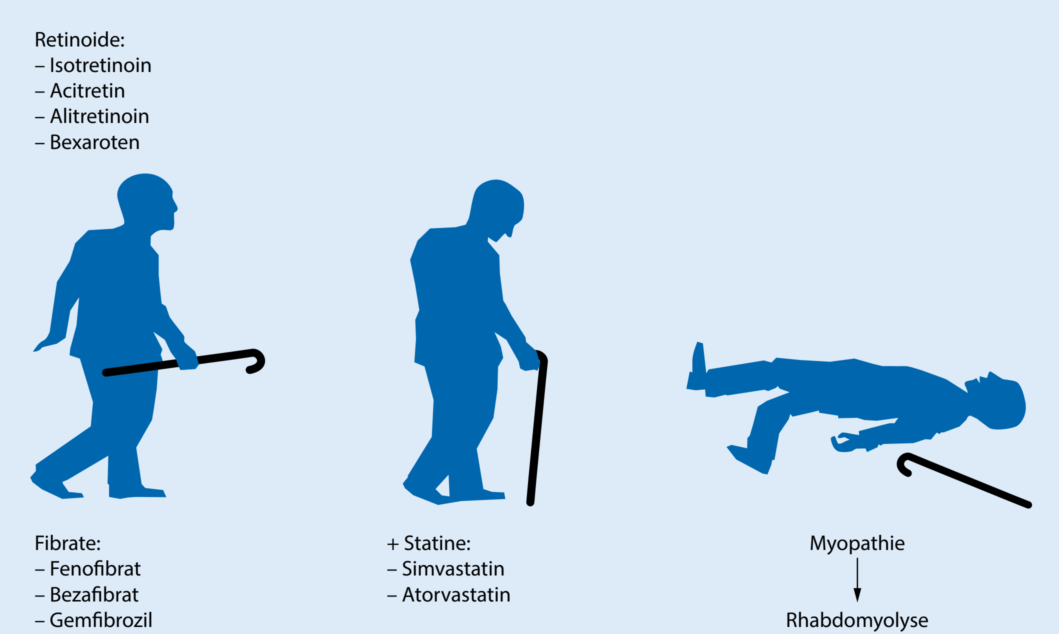
Retinoide:
– Isotretinoin
– Acitretin
– Alitretinoin
– Bexaroten
Fibrate:
– Fenofibrat
– Bezafibrat
– Gemfibrozil
+ Statine:
– Simvastatin
– Atorvastatin
Myopathie
Rhabdomyolyse

Online-Abb. 8

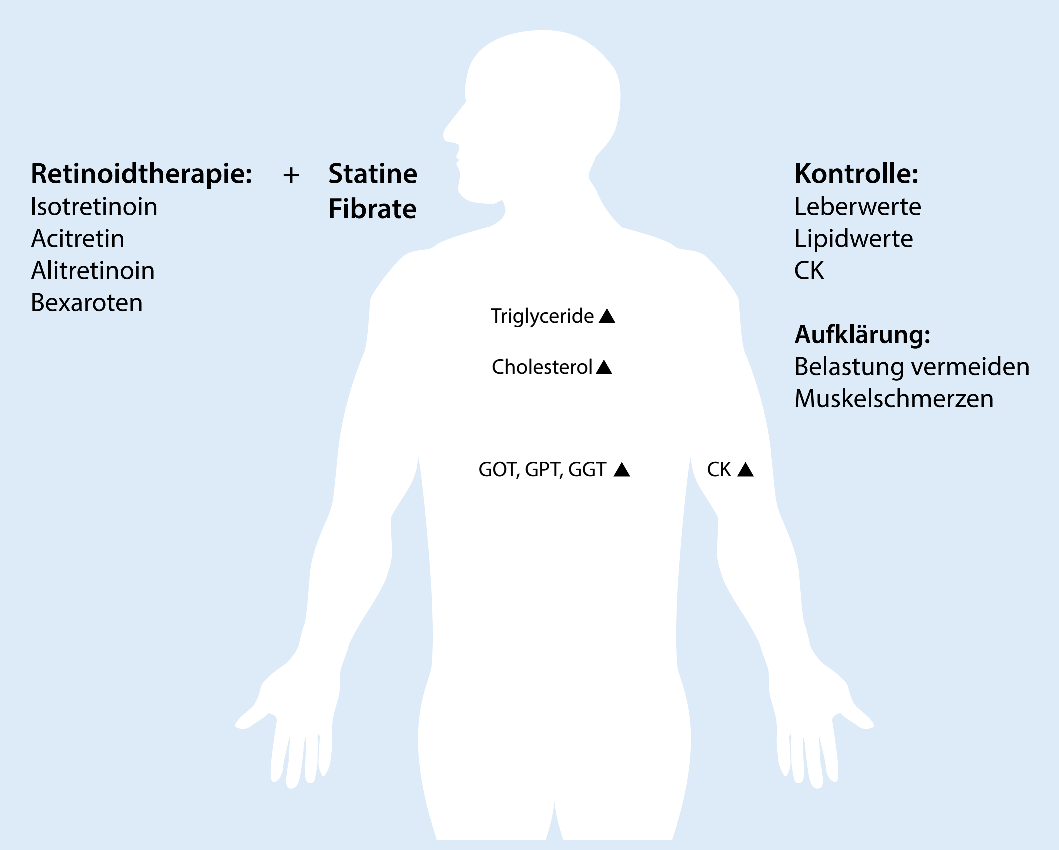
Retinoidtherapie:
Isotretinoin
Acitretin
Alitretinoin
Bexaroten
+
Statine
Fibrate
Triglyceride ▲
Cholesterol ▲
GOT, GPT, GGT ▲
CK ▲
Kontrolle:
Leberwerte
Lipidwerte
CK
Aufklärung:
Belastung vermeiden
Muskelschmerzen

Online-Abb. 9

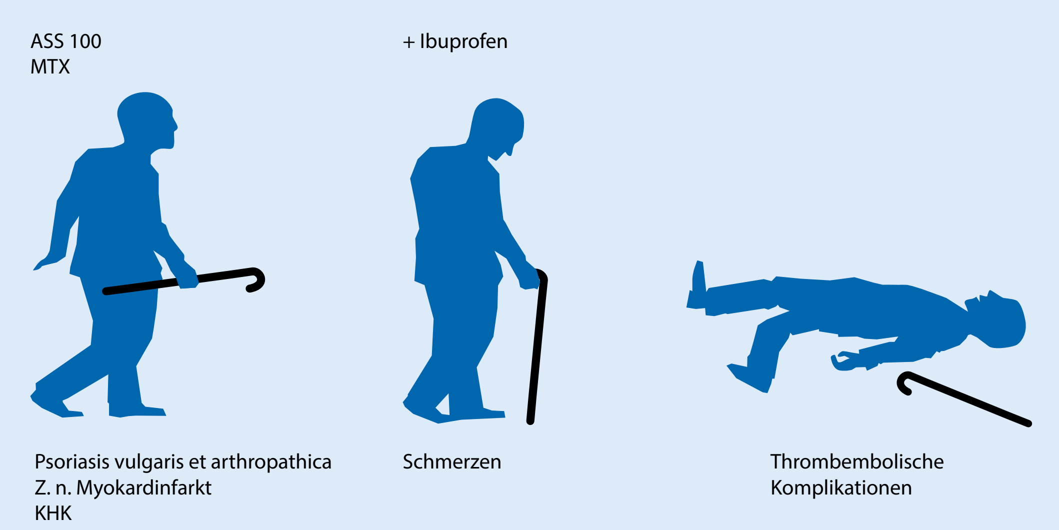

Online-Abb. 10

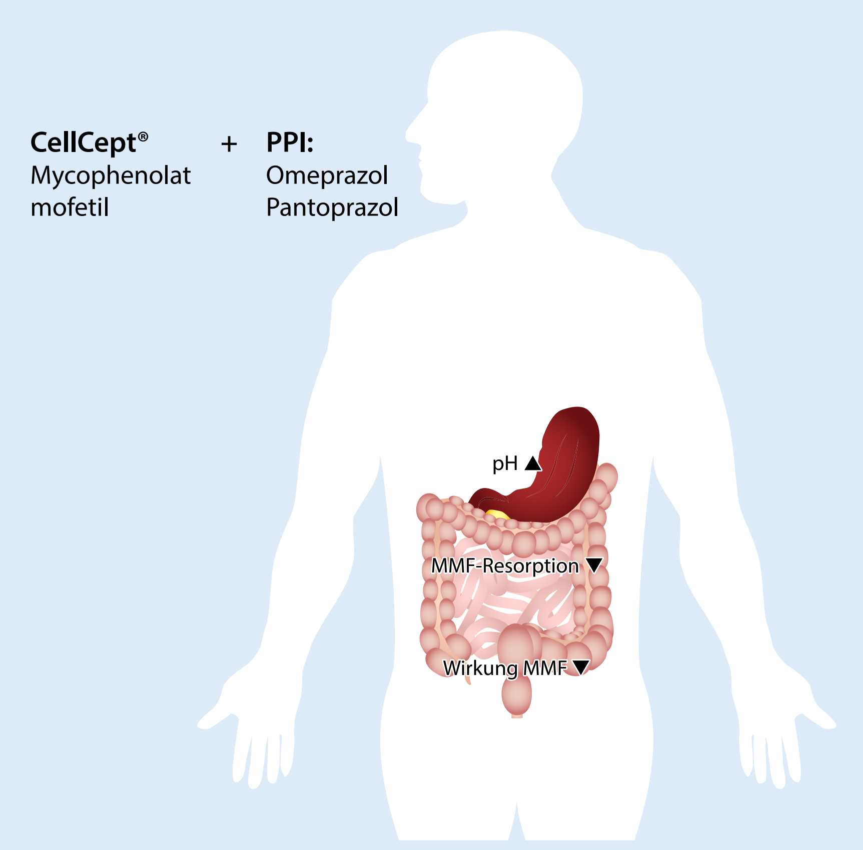

Online-Abb. 11

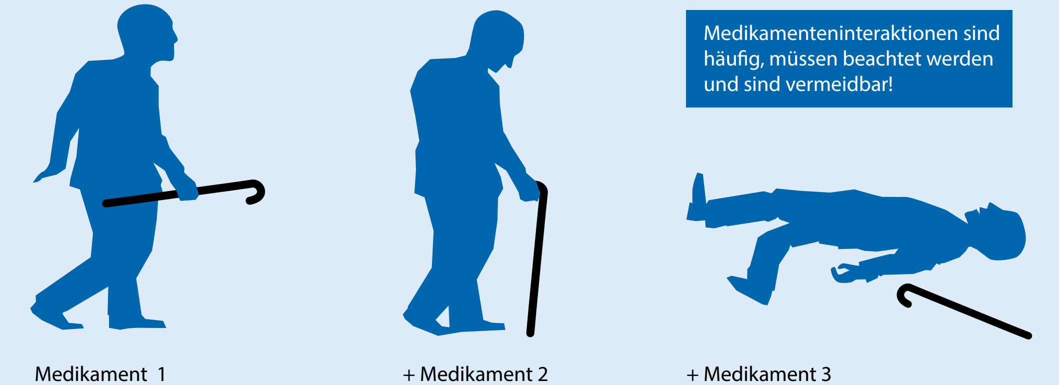

Online-Abb. 12
